# A case of eosinophilic annular erythema as a presenting sign for primary biliary cholangitis^☆^

**DOI:** 10.1016/j.abd.2023.05.014

**Published:** 2024-08-06

**Authors:** Pablo López Sanz, Noelia de Sande Rivera, Claudia Guerrero Ramírez, Silvia Manso Córdoba, Carlota Rodríguez de Vera Guardiola, Eduardo Escario Travesedo

**Affiliations:** a Department of Dermatology, University General Hospital of Albacete, Albacete, Castilla-La Mancha, Spain; b Department of Gastroenterology and Hepatology, University General Hospital of Albacete, Albacete, Castilla-La Mancha, Spain; c Department of Pathology, University General Hospital of Albacete, Albacete, Castilla-La Mancha, Spain

Dear Editor,

Eosinophilic Annular Erythema (EAE) is a rare skin condition that presents as a figurate erythema with an unpredictable clinical course. Whether it represents an isolated entity, or a variant of Wells Syndrome (WS) remains controversial, but its diagnosis is significant for its relationship with several systemic diseases and malignancies. We present the case of a patient presenting with EAE that led to the diagnosis of Primary Biliary Cholangitis (PBC), an association not reported in the literature.

A 62-year-old woman with a history of hypercholesterolemia consulted for intensely pruritic skin lesions that developed over the last month on her extremities and trunk. Additional history of new medications, changes in personal products, insect bites, and new sexual contacts was noncontributory. On systemic anamnesis, she referred intermittent abdominal pain centered on hypogastrium, asthenia, diarrhea and a non-intentional weight loss of 5 kg over the last year. Fever, choluria, or acholia were not present. Physical examination showed multiple erythematous annular plaques with elevated edges and central dusky hyperpigmentation on the lower and upper limbs and trunk (Fig. 1). Punch biopsies revealed deep and superficial perivascular lymphocytic infiltrate with numerous eosinophils and flame figures (Fig. 2), supporting the diagnosis of EAE. Laboratory tests were remarkable for positive anti-mitochondrial M2 antibodies at a 1:640 titer, cholestatic pattern (alkaline phosphatase, 108 UI/mL; gamma-glutamyl transpeptidase, 172 UI/mL; normal values of aspartate aminotransferase, alanine aminotransferase and bilirubin) and normal eosinophil counts. Abdominal and endoscopic ultrasounds ruled out mechanical bile duct obstruction, mass lesions and abnormalities of the gallbladder, leading to the diagnosis of PBC. The patient improved considerably on oral prednisone (30 mg/day) but experienced a flare when the medication was tapered. Ursodeoxycholic acid was initiated (900 mg/day) after the diagnosis of PBC without relapses of skin lesions and gastrointestinal symptoms after corticosteroid discontinuation and a 9-month follow-up. On laboratory testing, serum hepatic enzymes were also reduced to normal levels.

**Figure 1:**
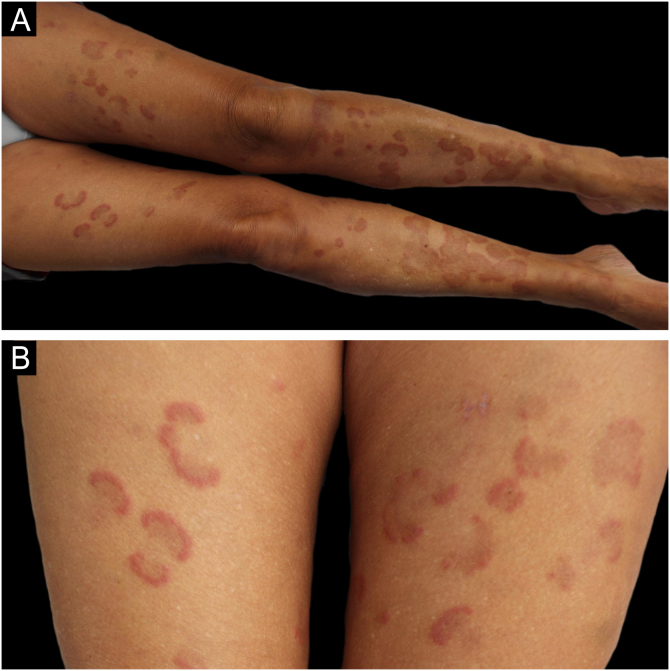
Annular and arcuate erythematous plaques with a dusky pigmentated center on the lower limbs (A). Detailed view of the lesions on the thighs (B).

**Figure 2:**
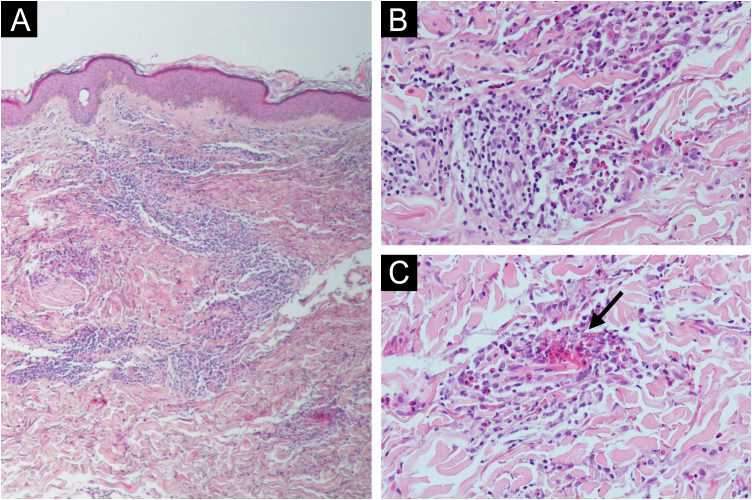
(A e B) Deep and superficial perivascular lymphocytic infiltrates with numerous eosinophils and flame figures (arrow) (C). Hematoxylin & eosin ×100 (A), ×200 (B‒C).

EAE is an eosinophilic, chronic, relapsing and remitting skin condition that clinically presents as urticarial papules and plaques on the trunk and extremities with slowly expanding arcuate or annular elevated edges leaving a pigmented center.^1^ Initially described in pediatric patients as annular erythema of infancy,^2^ it remains unclear if EAE represents a subset of WS (eosinophilic cellulitis) or a separate entity itself. Histopathologic findings include superficial and deep perivascular lymphocytic infiltrate with numerous eosinophils and, rarely, vacuolar degeneration of the basal layer.^3^ Even if it was believed that EAE could be differentiated from Wells syndrome by the absence of “flame figures”, multiple reports, including our case, have proven it wrong.^4^

When spontaneous healing does not occur, treatment is often effective with topical or oral corticosteroids and antimalarial drugs but relapses after discontinuation are common.^5^ Other treatment options reported in the literature are dapsone, specific cancer-associated treatment, methotrexate, anti-histamines, cyclosporine and minocycline. Isolated cases have successfully responded to dupilumab (IL-4 and IL-3 inhibitor) and benralizumab (IL-5 inhibitor) and this is not surprising since they are T-helper 2 type cytokines inhibitors, and a dysregulated tissue eosinophilia is thought to play an important role in EAE pathogenesis.^6^

EAE has been associated with thymoma, clear cell renal carcinoma, metastatic prostate cancer, autoimmune thyroid disease, borreliosis, chronic gastritis caused by Helicobacter pylori, diabetes mellitus, hepatitis C infection, chronic kidney disease, eosinophilic granulomatosis with polyangiitis, asthma, autoimmune pancreatitis and autoimmune hepatitis.^7^ Lower relapse rates and prolonged remission periods have been observed when these associated diseases are managed properly.^4^ To our knowledge, the association with PBC and EAE has not been reported.

Although the etiology of EAE remains unclear, it is suggested that it may be the result of a hypersensitivity reaction to an unidentified antigen.^8^ In the case of PBC, recent studies have demonstrated that serum CCL11, CCL24 and CCL26 are potent eosinophil-attractive chemokines that are up-regulated in PBC and could explain the possible association of PBC and the occurrence of eosinophilic dermatoses such EAE in our patient.^9^ In addition, the improvement of the PBC course and the resolution of skin lesion relapses with ursodeoxycholic acid provide extra support for this association in the present case.

In conclusion, we present a case of EAE that led to the diagnosis of PBC. Whether EAE is a distinct entity or not, its presence should raise awareness of an underlying systemic disease and a thorough patient work-up should be carried out.

## Financial support

None declared.

## Authors’ contributions

Pablo López Sanz: Study: Concept and design, data collection, or analysis and interpretation of data, writing of the manuscript, critical review of the literature, intellectual participation in the propaedeutic and/or therapeutic conduct of the studied cases.

Noelia de Sande Rivera: Study concept and design, critical review of the literature, intellectual participation in the propaedeutic and/or therapeutic conduct of the studied cases.

Claudia Guerrero Ramírez: Effective participation in the research guidance, intellectual participation in the propaedeutic and/or therapeutic conduct of the studied cases.

Silvia Manso Córdoba: Effective participation in the research guidance, intellectual participation in the propaedeutic and/or therapeutic conduct of the studied cases.

Carlota Rodríguez de Vera Guardiola: Data collection, or analysis and interpretation of data, effective participation in the research guidance.

Eduardo Escario Travesedo: Effective participation in the research guidance, intellectual participation in the propaedeutic and/or therapeutic conduct of the studied cases, final approval of the final version of the manuscript.

## Conflicts of interest

None declared.

## References

[1] Heras M.O., Muñoz N.P., Sancho M.I., Millet P.U. (2017). Eosinophilic annular erythema in adults: report of two cases and review of the literature. An Bras Dermatol.

[2] Fania L., Provini A., Rota L., Mazzanti C., Ricci F., Panebianco A. (2020). Eosinophilic annular erythema: report of four cases. Dermatol Ther.

[3] Kim Y.S., Song Y.M., Seo H.M., Bang C.H., Lee J.H., Lee J.Y. (2017). Eosinophilic annular erythema associated with churg-strauss syndrome. Ann Dermatol.

[4] El-Khalawany M., Al-Mutairi N., Sultan M., Shaaban D. (2013). Eosinophilic annular erythema is a peculiar subtype in the spectrum of Wells syndrome: a multicentre long-term follow-up study. J Eur Acad Dermatol Venereol.

[5] Chastagner M., Shourik J., Jachiet M., Battistella M., Lefevre G., Gibier J.B. (2022). Treatment of eosinophilic annular erythema: retrospective multicenter study and literature review. Ann Dermatol Venereol.

[6] Moro-Bolado F., Martínez-Montalvo L., Al-Wattar-Ceballos O., Galindo-Bonilla P.Á., García-Arpa M. (2023). Treatment of eosinophilic annular erythema with benralizumab. JAMA Dermatol.

[7] Awosika O., Totoraitis K., Eleryan M., Rengifo-Pardo M., Ehrlich A. (2017). A case of eosinophilic annular erythema as a presenting sign for autoimmune hepatitis. JAAD Case Rep.

[8] Prajapati V., Cheung-Lee M., Schloss E., Salopek T.G. (2012). Spontaneously resolving eosinophilic annular erythema. J Am Acad Dermatol.

[9] Lin F., Shi H., Liu D., Zhang Z., Luo W., Mao P. (2019). Association of CCL11, CCL24 and CCL26 with primary biliary cholangitis. Int Immunopharmacol.

